# Book Reviews

**Published:** 1807-01

**Authors:** 


### CRITICAL ANALYSIS

### OF THE

### RECENT PUBLICATIONS

### ON THE

### DIFFERENT BRANCHES OF PHYSIC, SURGERY, AND MEDICAL PHILOSOPHY.

*Practical Observations on the principal Diseases of the Eyes, illustrated with Cases.* Translated from the Italian of ANTONIO SCARPA, Professor of Anatomy at the University of Pavia. F. R. S. Lond. and Member of several other learned Societies, with Notes. By JAMES BRIGGS, Member of the Royal College of Surgeons of London, and Assistant Surgeon to the Public Dispensary. 8vo. London, 1806.

[ Concluded from vol. xvi. pp. 562—568. ]

The subject of Ophthalmia is continued through all its varieties, and the remedies best suited to each, are amply explored.

The 8th Chapter is on that cloudiness of the cornea which often follows obstinate chronic Ophthalmia. This, which our author calls the nebula of the cornea, is accurately distinguished from that dense and dark * shot, which he denominates leucoma or albugo. The disease is imputed to a relaxation of the blood vessels, in consequence of which they become varicose, increasing in size and number. This expression of relaxation is a very uncertain one. It would be more consistent with what we have now discovered of diseased actions to suppose, that in this instance it had

* We have not seen the original, but suspect that the word here transslated *dark*, should rather have been *opake*.

had become habitual, as we find the case in that chronic inflammation which gave rise to it. This is, however, of less consequence, since the practice would, in both cases, be similar. First, as our author proposes, by what he calls astringent and corroborant remedies, to induce a contraction in the vessels themselves; and if that should not succeed, to cut through, or if necessary, cut out the vessels themselves; the last we think may be avoided, by causticating the cut edges of the vessels. The first remedies, more probably, act by a stimulating property, by which they excite new actions in the part, which will sometimes end in restoring the original actions; if this does not succeed, the proposed operation often produces a cure.

The next chapter is on the Albugo, and Leucoma. The remarks are very judicious, but nothing new occurs. The succeeding chapter, particularly on ulcers of the cornea abounds with highly useful practical observations.

Chapter xi. treats of the Pterygium, which the Professor defines "a small preternatural membrane, of a reddish ash colour, and of a triangular figure [acquiring its name from some resemblance to a wing], which arises in general from the internal angle of the eye, near the caruncula lachrymalis, and extends gradually upon the cornea, attended with considerable injury to the sight." This he has proved by dissection (well illustrated in a plate), is nothing more than a morbid thickening of the conjunctiva. The further description and mode of treatment are well explained, and judiciously stated.

The 12th Chapter contains some very useful remarks, practical and physiological, on the Encanthis, or excrescence arising from the caruncula lachrymalis, and the mode of detaching it with the greatest safety.

The 13th chapter is on Hypopion, or the occupation of one or both chambers of the eye, with a glutinous opake fluid, instead of the true transparent humours. The author considers this as the sequela of an acute inflammation, and proposes, as the principal and safest mode of cure, whatever will stimulate the absorbens. In considering the nature of the disease, he slightly touches on the opinion of the Hunters, concerning the secretion of matter without ulceration; we say slightly, because we think, on this occasion, Professor Scarpa, whose abilities we respect, as much from our personal knowledge as from his writings, might have used a more decided language on this occasion. He seems unwilling to admit matter without ulceration, or rather without a breach of the solids. "If, however, says he, it should be insisted, that there is no essential difference between coagulable lymph effused from a membrane violently inflamed and *matter*, it must be conceded that there are cases in which matter is formed without abscess or ulceration, and that the hypopion is a disease of this description."

By

By this passage it is evident how little Mr. Hunter is understood, even by this most candid enquirer. High inflammation would only throw out coagulable lymph even on a secreting surface; but as this high inflammation subsides it may end in secretion of matter, as we find the case in the urethra and other such membranes. Now Prof. Scarpa is old enough, and has opportunities enough, not to leave this as matter of question, and his language ought to be more decided, for by the decision drawn from experiments, conducted by teachers of this decription, can we only expect to arrive at truth.

It is, however, but justice to add, that we highly approve of his mode of treatment. Puncturing the cornea, merely because the contents are purulent, is certainly a dangerous, and for the most part unnecessary practice; and it does great credit to the writer, that, regardless of all authorities in practice, he opposes established opinion wherever he sees it necessary.

Chapter xiv, Is of *Procedentia Iridis.* This can only happen when the cornea is punctured or ulcerated, in which cases the iris must, if the aperture continues open, be gradually protruded. We confess we have not seen enough of this disease to offer any remarks of our own, we shall therefore refer the reader to the work itself for every information he may wish on the subject.

The succeeding Chapter is on Cataract. As this is now more a subject of controversy concerning the mode of cure, than the nature of the disease, and as we doubt not our chirurgical readers have perused every thing of consequence on the subject which has appeared in England, we shall content ourselves with stating Professor Scarpa's opinion.

" There are two methods of treating the cataract, the one by removing the opake crystalline, from the visual axis of the eye, by means of a needle: the other, by extracting it from the eye, by making a semicircular incision in the base of the cornea.

" It has long been disputed which of these two methods ought to have the preference: and in the warmth of discussion, the advantages of the one, and the disadvantages of the other, have been exaggerated by both parties. Observation and experience, however, the great teachers in all things, seem to have pronounced in favour of the ancient method of treating the *cataract*, or that of *depression*; not only because *depression* is more easily executed than *extraction*, and can be equally employed in every species of cataract, whether crystalline or membraneous, solid or fluid; but because *depression* is attended with symptoms far less violent and dangerous than those which very frequently happen after extraction; and if from any accidental cause this operation should occasionally prove unsuccessful, it may be repeated two or three times upon the same eye without any risk; a circumstance which *extraction* does not admit of, when that operation has not had the desired success.

"Influenced by these facts, I have for a considerable time laid aside the method of treating the cataract by extraction, and have applied myself entirely to the practice of depression, and I see continually great reason to be satisfied with the choice which I have made. The very frequent occasions which I have had of performing this operation, have afforded me an opportunity of making some useful alterations relative to the means which are employed previously to its execution; of which I shall now proceed to give a detail.

"It is easy to determine whether the operation can be performed with a prospect of success or not. A favourable issue may be expected, whenever the cataract is simple, or without any other disease of the eye-ball, in a subject not quite unhealthy or decrepid, and in whom the opacity of the crystalline humour has been gradually formed, without having originated from any external violence, or habitual ophthalmia, especially the *internal:* where there has not been frequent pain in the head, eye-ball, and supercilium: where the pupil, notwithstanding the cataract, has preserved its free and quick motion, as well as its circular figure, in different degrees of light: and lastly, where, notwithstanding the opacity of the crystalline lens, the patient retains the power, not only of distinguishing light from darkness, but also of perceiving vivid colours, and the principal outlines of bodies which are presented to him, and where the pupil has that degree of dilatation which it is usually found to have in a moderate light."

Several other remarks follow, some of which we think might have been omitted, and the author might have proceeded at once to the absorption of the parts which gave rise to the operation. Were it not for this, we suspect all his cautions relative to the manner of disposing of the lens and its capsule would often be of no avail. But it is not a little curious to see how extremely difficult it is even for Professor Scarpa to bring his mind to the absorption of solid matter. He first speaks of the dissolution of the capsule; afterwards of its gradual liquefaction. Why does he not make the experiment of leavng part of the capsule immersed in the fluids of a dead eye, and see whether those fluids have a power of dissolving it, or whether those changes, which he so accurately traces in the supposed progress of liquefaction, will take place any where but in a living eye?

This hapter seems to us extended to an unnecessary length; but considering the delicacy of the operation, it is probable tha the practical occulist may be of a different opinion.

Chapter xvi. On the artificial Pupil. This the author has sometimes found a necessary operation, in consequence of the aperture of the iris being closed after the depression or extraction of the cataract, and also after violent inflammation.

A Chapter on Staphycoma follows, containing a good definition of the author's appropriation of the term, and some very useful practical remarks.—Next follows

A Chap-

A Chapter on the Dropsy of the Eye, another on Amaurosis and Hemerolopia, or periodical blindness, and the last on calculous Concretions of the Eye. On all these we meet with very useful practical remarks; but having already, on account of the variety of matter, extended this article beyond our usval limits, we must conclude with congratulating the practical surgeon on a very valuable addition to this branch of surgery, and recommending the work as a necessary part of the library of every provincial practitioner, who has not those frequent opportunities of improving his knowledge, which the metropolis or larger cities afford.

---

MALVERN WATERS, *being a Republication of Cases, formerly collected by* JOHN WALL, M. D. *and since illustrated with Notes, &c. by his Son,* MARTIN WALL, M. D. *&c. &c.*

THE importance of those remedies which nature offers to us, elaborated in the bowels of the earth, are not to be estimated by their more obvious properties. Malvern Waters are generally admitted to be purer than those of any other spring, yet many circumstances show that they possess properties, and therefore, probably ingredients not to be found in other waters, nor to be imitated by art.

The present publication contains the late Dr. Wall's inquiries on these subjects, much enriched by the addition of Dr. Martin W's remarks. The improvements in chemistry, which have taken place, induced Dr. Wilson to offer his analysis of these waters. This last circumstance rendered a republication of this work, in its present state, more desirable, as containing many practical remarks, well calculated to render the united labours of the three, as complete an account of the waters as the present state of chemistry will admit.

Of a work of this kind 'tis of little use to offer an analysis. Suffice it to say, that the number of well authenticated cases are sufficient to encourage the faculty in recommending a trial in all those chronic, ill conditioned ulcers, which are usually called the worst degrees of scrofula. The following passage we have selected, as containing one of many striking cases, and some very useful remarks.

"The waters, upon their first use, in some create a slight nausea; others they purge briskly for three or four days; but are diuretic in all. The former effects are probably accidental; arising only from their being taken in too large quantities, or from bilious foulnesses in the first passages. Persons who have been much used to malt liquors, they commonly render costive: but there are instances, where the waters, after having been drunk a considerable time, suddenly take a turn downwards and purge briskly. Such an effect they had upon the late Edward Popham, Esq. of the Lodge near Tewkesbury. This gentleman was crippled by the gout, and had in a manner lost the use of all his limbs. The marvellous cures performed at the holy well induced him, about ten years ago, to make a trial of it. After he had drunk the water about a month, a violent diarrhœa came on, and lasted several days, from which he found great relief; recovering his spirits, and in a great measure the use of his limbs. As a testimony of the benefit he had received, he built a bath under the spout, the ancient one being in a very ruinous condition.

"I always advise my patients to drink freely of the water for some days or weeks before they use them externally. The empirical method of application, which has hitherto been successfully practised, is to wash sores, tumours, &c. under the spout several times in a day; covering the parts afterwards with cloths dipped in the same water, and moistened from time to time as often as they grow dry. Those who bathe also for cutaneous foulnesses, usually go into the water with their linen on; and dress upon it wet. This method, odd as it is, has never yet, as I have heard, been attended with any ill consequences; though I have known it used by several very tender persons.

"Dr. Heberden has mentioned this circumstance, with others, to prove that our fears of damp linen, damp sheets, damp houses, &c. are not founded on attentive observation. (See *Medical Transactions*, vol. ii. p. 525.) But, with all due respect to so great a name, it may be doubted, whether this or any other argument, which he has adduced, is sufficient to overthrow an opinion almost universally received: as most persons are unfortunately able, from their own experience in some period of their life, to add their testimony of its truth." M. W.

We have given this passage as a motive for further enquiry on an important question. Dr. M. W's remark, is by no means a satisfactory answer to Dr. Heberden's judicious enquiry. Many popular errors are as universal as the present opinion: besides which, it does not appear to have been the wish of the ingenious enquirer to assert, that there are no circumstances under which damp linen may be injurious, but that the general terror of it is unfounded.

---

*Observations on the Nature, Kinds, Causes, and Prevention of Insanity.* By THOMAS ARNOLD, M. D. Fellow of the Royal College of Physicians, and of the Royal Medical Society of Edinburgh; in Two Vol. Second Edition, corrected and improved, 1806, London.

OF all the physical calamities to which man is liable, madness is often considered as the most humiliating. Yet, if we view the question with accuracy, perhaps we shall say, that there is scarcely an event which so strikingly distinguishes the human from all other animals. When we see an animal incapable of associating ideas with objects, or of combining impressions, so as to form conclusions which are to determine his actions; and consider this condition as a peculiar exception from the rest of his race, we may surely be proud of being numbered in such a class.

But that sympathy, which is interwoven with our very existence, excites sentiments, which absorb all others in the presence of such an an object. If reason is our distinguishing faculty, how dreary the sight of a human being divested of such a distinction. If, as is often the case, such an object is not only insensible to his own situation, but even proud of a fancied superiority over those around him; let us, under all these circumstances, improve by the lesson before us, and unite with our sympathy those reflections which may render us grateful for what we possess, and humble from the uncertain tenure on which we hold it.

On so obscure a subject, we must be thankful for whatever is offered us. The ancients were particularly imperfect, even in their conjectures on insanity. Their little knowledge of anatomy, and that little derived principally from the dissection of brutes, precluded them from experiment, the foundation of all rational investigation. Among the moderns, little is to be learned before our own times. Battie has the merit of attempting something, and had he been treated with more candour by his rival, might, probably, have improved his reasoning, as his judgment was matured by experience. Dr. Arnold has taken a more comprehensive view of the subject, and attempted some arrangement superior to that of general nosologists, in proportion as his views were particularly directed to the subject. Dr. Crighton wished to improve the plan, but with fewer opportunities, his performance has been proportionally imperfect. Mr. Haslam has offered an admirable practical work, which, it is much to be regretted, that he has not further improved.

The valuable work before us, begins with a preface to the second edition, in which, the author vindicates his arrangement from the objections made to them by Dr. Crighton. It must be confessed this is not a very difficult task. It would have been more important to show the necessity of those distinctions which so often encroach on each other; but as Dr. C. has divided, distinguished, subdivided and *sub-distinguished*, to an almost endless, and some will say, useless minuteness, he was not the person who should object to the few species proposed by our author, all of which are included in one genus.

We would not be thought to express an entire aversion to divisions, even though they cannot always be well maintained. They oblige the mind to arrange, combine and associate, and lead to enquiries which might otherwise have been neglected. But when they have no better foundation than metaphysics, or when they lead to no practical inferences, we cannot help regretting the misapplication of industry which might be much better employed. In a word, we are ready to consider our author's nosological divisions in no other light, than as necessary to assist arrangement in the descriptive part of his performance; and in this manner, it appears his wish to be understood, by superscribing the chapter *definition and arrangement*. That we may therefore avoid doing injustice to a scheme, which cannot be well comprehended, without a careful perusal of the whole, we shall offer only the outlines of the general arrangement.

Every definition of insanity, from Hippocrates to Bishop Berkely, is carefully and candidly examined, and to do our author justice, we find marks of industry and accuracy which do ample justice to his head and heart. But we regret much, that he has not selected a proper place for his own definitions, unconnected with any authorities. With the advantages of Dr. Arnold, the result of his observations would have been sufficient, without the support of Von Helmont, or any other of the venerable tribe, quotations from whom, rather interrupt than illustrate the subject. The latter might have served as an historical account of the opinions concerning madness, to introduce the more correct definitions and arrangements of the author.

The following is the table of insanity.

"*A Table of the Species of Insanity.*

"*One Genus,* Insanity. *Two Divisions,* Ideal—and—Notional.

"I. Ideal Insanity.

"1. Phrentic. 2. Incoherent. 3. Maniacal. 4. Sensitive.

"II. Notional Insanity.

"5. Delusive. 6. Whimsical. 7. Fanciful. 8. Impulsive. 9. Scheming. 10. Vain, or self-important. 11. Hypochondriacal. 12. Pathetic. 13. Appetitive."

The general definition and the discriminating marks of the two divisions had been giving in a previous passage. We shall select it, as well as we can, from the quotations with which it is intermixed.

"The faculty of perceiving material objects, and their grosser qualities, by means of the senses, we possess in common with brutes: but the power of comparing their several relations and properties, and of reasoning analogically concerning them: the power of abstraction; and that reflex action of the mind by which it is enabled to review its internal treasures, and to contemplate its own faculties and operations; which lead to the discovery of almost an infinity of new truths and probabilities, and are the inexhaustible sources of every species of knowledge; are, in a great measure, the exclusive privilege of man.

"About the former it is obvious that the mind can err, in any considerable degree, only by some defect in the bodily organs, whether natural or acquired, permanent or transient.

"About the latter it may err from a variety of causes; which might all, perhaps, not unaptly be arranged under the following heads:—a natural incapacity, or habitual deficiency of attention,—weakness of memory,—too great activity, and indulgence of imagination,—depravity of will,—excess of passion, which is the natural consequence of them all—and disease of body.

"These errors may be very considerable, and unreasonable, without out constituting madness:—to deserve that appellation, they must appear of a certain magnitude, and under certain circumstances and limitations, which I shall now proceed to point out. It must, however, be acknowledged, that it is frequently difficult, especially with regard to the latter sort of mental errors, exactly to define where folly ends, and insanity begins.

"The mind may be said to be delirious when it supposes sen-"sible objects to exist externally, which exist, as they then ap-"pear to the mind, only in idea:—or as such notions about ob-"jects which it sees, hears, or otherwise perceives, or knows, "as appear obviously false, or absurd, to the common sense and "experience of the sober and rational part of mankind.—De-"lirium, therefore, may naturally be divided into two kinds:—"the one arising from an error in our ideas, I call *ideal delirium*; "and the other, arising from an error in our notions, I call *no-"tional delirium.*"

"The former kind of delirium is common both to fever and madness;—the latter, I am inclined to believe, is peculiar to madness,—and suspect that whenever any degree of it is to be observed in the delirium of a fever, it portends that it will probably end in madness.

"That the delirium of the true phrensy, and other high degrees of febrile delirium, are of the ideal kind, is obvious to the most superficial examiner; but that the slighter degrees, which chiefly affect the patient, on first waking out of sleep, with absence, muttering, wandering, rage, or terror; which greatly abate, or cease altogether, as the remaining effects of sleep are dissipated; are all likewise of the ideal kind, is not at first view so obvious: but upon a stricter scrutiny, we may perceive that these symptoms arise chiefly from delirious images in the brain, which being but slightly improved, while the brain is but slightly affected, are only vivid during sleep, which shuts out the glare of external objects; and gradually vanish as sleep gives place to waking; just as dreams of children often continue for a while after they are apparently awake, their senses being with difficulty roused, and drawing off the attention by slow degrees from the ideal picture presented during sleep, to the real representation of surrounding objects.

"If what has been advanced be just, I may now, with some degree of clearness and precision, proceed to define insanity; and to enumerate and describe such of its species as have fallen under my own observation, or have been noticed by other medical writers.

"Isanity, as well as delirium, may be considered as divisible into two kinds; one of which may be called *ideal*, and the other *notional insanity*.

"Ideal Insanity is that state of mind in which a person "imagines he sees, hears, or otherwise perceives, or converses "with, persons or things, which either have no external existence "to his senses at that time;—or have no such external existence

"as they are then conceived to have;— or, if he perceives exter-"nal objects as they really exist, has yet erroneous and absurd "ideas of his own form, and other sensible qualities;—such a "state of mind continuing for a considerable time; and being "unaccompanied with any violent or adequate degree of fever."

"Insanity of this sort is sometimes attended with fear, sometimes with audacity, sometimes with neither; and may be either constant,—remittent,—or intermitent.—The constant has no very observable, nor any regular remissions: the remittent usually grows milder once in twenty-four hours, generally in the day-time, and has exacerbations in the evening;—the intermittent has considerable lucid intervals; and as the paroxysms of this sort of madness have been commonly supposed to obey the full and change of the moon, it has therefore been peculiarly distinguished by the name of *lunacy;*—a name which has, however, been indiscriminately extended to every species of insanity.

"NOTIONAL INSANITY is that state of mind in which a per-"son sees, hears, or otherwise perceives external objects as they "really exist, as objects of sense; yet conceives such notions of "the powers, properties, designs, state, destination, importance, "manner of existence, or the like, of things and persons, of him-"self and others, as appear obviously, and often grossly errone-"ous, or unreasonable, to the common sense of the sober and "judicious part of mankind. It is of considerable duration; is "never accompanied with any great degree of fever, and very often "with no fever at all."

"Notional, like ideal insanity, may be either with or without fear or audacity; it is usually constant;—but in some cases it remits—and even intermits,—though for the most part with great uncertainty and irregularity.

"Insanity is easily distinguished from the temporary and transient delirium of intoxication, whether occasioned by wine, opium, or any other inebriating substance,—from the delirium which sometimes accompanies hysteric fits,—and others of a like nature; —not by a knowledge of the cause, but by the duration of the delirium;—for even the delirium arising from any of these causes becomes insanity, if it continue long after the original exciting cause hath ceased to act. Thus intoxicating substances may not only produce transient delirium, as is usually the case; but sometimes, either in a brain predisposed to insanity, or when taken to great excess, or when the intoxication has been frequently and habitually repeated, their pernicious effects may be more permanent; and indeed it too often happens that actual madness, of various kinds, as circumstances may chance to determine, is the dreadful consequence of this kind of intemperance."

On this passage we shall make a few remarks, some of which may probably be borrowed from the work before us. We would gladly give the author credit for them in his own words, could we make such an extract as would convey our united meaning without interruption interruption by those authorities, which we have before remarked, are so ill placed.

We must first carefully distinguish that state of the mind in which the senses are torpid, but the recollection of former objects presents itself without our being able to distinguish them from realities. When this is attended with an incapacity of muscular action it is called sleep; when the senses only are torpid it is called reverie; and when this reverie induces impressions strong enough to excite corresponding muscular actions, we then term it Somnambulism. The two last arise from that state of the senses which periodically affects every part of the body whose actions are not necessary for the support of life, and the only peculiarity in them, is, that in these instances there is an irregularity in the quiescent periods of parts which are usually torpid at the same time. But in both these cases, as well as in sleep, any stimulus inducing a sufficient impression on any of the senses, restores the susceptibility of all the rest, and the man awakes, recovers from his reverie, or ceases from that train of actions to which he was previously impelled by objects which only existed in his own imagination.

Such a temporary state is easily distinguished from one in which the mind is permanently incapable of associating ideas corresponding with the objects perceived by the senses, or of drawing probable inferences from the sight and knowledge of such objects, or of drawing probable conclusions from a combination of events, the nature and relations of which it is capable of comprehending, or, lastly, whenever it is incapable of tracing the absurdity of postulates, yet as willing to admit the validity of every induction as if the postulate were a thing which would admit of no dispute. Under all these circumstances we may say such a mind is deficient, and such deficiency must arise from original formation, from wrong action induced by some other temporary cause, or from a real change in those organs which enable us to form combinations or conclusions. If the deficiency is original or congenital, it is usually called Idiotcy or imbecility. If the effect of a temporary cause, Delirium. If unconnected with any other apparent change, Madness.

When madness shows itself by an incapacity to associate objects with corresponding ideas, it must either arise from the rapidity of all the actions of those parts which are impressed by the objects of sense, or from so strong an impression already imposed, as to prevent the effects of impression from all others. The first is usually attended with violent symptoms called phrenzy, the latter either with the deepest melancholy, or with such a fondness for certain objects that the mind seems wholly engaged in an agreeable illusion. In either case, if such a state remains permanent, we can have no doubt in calling it madness.

But when we have no other proof than an incapacity of making just combinations, drawing probable inferences, or reasonable conclusions, we must consider the character of the person, or compare his present state with what we have witnessed in him on former occasions.

occasions. If the deficiency is so great that the person seems incapable of attending to self-preservation, and at the same time shows a wish to live, and on former occasions avoided dangers of which he is now unconscious, no one will question the state of such a man. But if in a longer chain of reasoning he should obstinately persevere in error, and support that error by arguments, the fallacy of which by the closeness of his reasoning we might expect he must be aware of, we may fairly suspect the soundness of such an intellect, and this suspicion will extend to all the different shades of design, partiality or absolute insanity, according to the character of the person, the degree of error, and the various other circumstances and impressions under which he has formed his conclusions.

We are aware it will be said, that if this is to be the test of madness, not only *nemo omnibus horis sapit*, but *nemini omnibus horis est mens sana*. We must therefore carefully make a distinction between occasional aberrations and the *omnibus horis*, as well as the degree of intellect in the person. That is, we must make allowances for the passions and folly of individuals. When Lord Baltimore tells Miss Woodcock that there is no God, we impute such an assertion to a base mind. When the young woman replies that she can prove he is wrong *from Scripture*, we consider such an answer made to such a person as the effect of folly or imbecility. But when a learned and able reasoner, and disinterested man, attempts to defend the errors of a madman, who fancies himself the nephew of Jesus Christ, we cannot help suspecting such an incapacity to discover the fallacy of a first principle as absolutely inconsistent with such powers of reasoning, and have only one way of accounting for what we hear or read.

Such appear to us the general divisions of lunacy. The various turns and dispositions in different individuals will resolve themselves into one of these. The only necessary distinctions beyond them, are in our opinion applicable to all the varieties, and may be divided into chronic, acute, habitual, and periodical. Such seem to us the only divisions which can lead to practical inferences; all beyond must be governed by the immediate symptoms, and the previous history of each individual case.

Having said thus much, we shall only be expected to give a cursory view of the subdivisions of the above table. Under the pathetic, our author includes amorous insanity, jealous, avaricious, mysanthropic, arrogant, irascible, abhorrent, suspicious, bashful, timid, sorrowful, distressful, nostalgic, superstitious, fanatical, and desponding. Some of our readers will complain of this unnecessary and minute division. It will however be more candid to thank the author that he did not increase the number, for when he got thus far, a very transient view of his dictionary might have helped him to as many as would have occupied another volume in enumerating and defining them.

A chapter concerning the appearances on dissection, concludes the volume. These are taken from Bonetus, Morgagni, and Haller. We

We regret that this industrious and acute observer has made no addition from his own obserations.

The second volume contains the causes of insanity and the means of prevention. Under the first head, the author thinks it unnecessary to include the *predisposing*, by which we conceive he means the hereditary, cause. It may be said that this is irremediable, but so are many other causes which it has been found useful to investigate, and in our opinion it is always much more desirable to meet every evil, and see its full force, instead of carefully passing it over. Hereditary diseases lose much of their terror when accurately enquired into. It is found that the disposition wears out in all families, and in the greater part of the individuals is never brought into action. It is also to be recollected, that a solitary instance in a family is no proof of an hereditary disposition.

The remote causes are divided into bodily and mental. The first comprehends, 1. Causes seated in the brain, its vessels or membranes. 2. External causes, which operate mechanically, or at least immediately on the brain, as compression from any cause, concussion, or coup de soleil. 3. Causes affecting the body in general, which produce insanity either by acting directly upon the brain, or by introducing a gradual change in the system, which disposes to insanity. These last comprehend acute diseases in general, with excessive evacuation, inanition, or inactivity. 4. Diseases in various organs producing a sympathetic effect on the brain; these comprehend almost every disease, and almost every species of irritation to which the constitution or parts are liable.

Mental causes have only four divisions, but one of the sub-divisions is sufficiently numerous.

"Mental Causes.

"V. Intense application of the mind to, 1. Study, 2. Business, or schemes, of any kind, that require great and unremitted attention, or much exertion of genius; or, 3. Any sort of employment of the mind, which may keep it for a long time in an active and wakeful state.

"VI. Passions of various kinds, when sudden, violent, or habitual; as, 1. Surprize, or astonishment. 2. Joy. 3. Enthusiasm, or religious joy. 4. Pride and vanity. 5. Desire. 6. Anger. 7. Hatred and aversion. 8. Love. 9. Ambition. 10. Avarice. 11. Distress. 12. Grief. 13. Despair. 14. Fear. 15. Suspicion. 16. Jealousy. 17. Anxiety. 18. Religious fear.

"VII. Too great activity of imagination.

"VIII. Imbecility of mind."

We shall make no comments on this passage, but leave our readers to judge what advantage is to be derived from the enumeration of these passions. In commenting, however, on these various causes, the author shows much judgment, considerable reading, and a readiness at producing authorities, not only from medical writers but from all the classical authors in both languages. In such a variety as is to be met with, it is difficult to form any selection tions, we hall however transcribe the following passage as containing an important fact, which, though long since established, has been too much overlooked.

"But tumultous, excessive, and irrational joy, is as injurious to the body and mind, as a just and grateful satisfaction is salutary to both. It produces a sudden increase of tone, and irritability, a quick and often irregular circulation, and a general determination to the surface of the body; an increase of perspiration, and other secretions, of heat, and of colour; a peculiarly increased activity of the vessels of the head and brain, a highly active state of imagination, great heat of the head, and flushing of the face. From the increased irritability, from the quick succession of pleasing ideas, from the accelerated motion of the small vessels of the head and brain, it is usually attended with benevolence and generosity, and often with tenderness, and tears; and if sudden, violent, or durable, has been known to produce fainting, fevers, insanity, and sudden death.—In producing insanity it seems to be not less powerful than even grief. And it is remarkable that, as we are told by Dr. Mead, on the authority of Dr. Hale, who was at that time physician to Bethlehem-hospital, that of the great number of persons who became insane in consequence of their connexions with the South-sea Company, in the year 1720, there was a much larger proportion of those successful adventurers whom fortune had favoured with the sudden acquisition of immense riches, than of those who were completely ruined by that iniquitous imposition."

It is a most important consideration, that the human mind is not only improved by adversity, but that the sensations excited by it are less apt to disorder the frame than those which attend prosperity. It is not difficult to see the reason of this. Under adversity the mind feels its own weakness as well as its own powers; it feels a consciousness of the necessity of exerting the latter. When properly directed, it never fails to meet with a corresponding assistance from others, and is usually cheered by hope, or consoled by reflections on the uncertain tenure of all human possessions.—In prosperity, our prospects lengthen as we rise; forgetful of those casualties to which we owe our exaltation, we conceive the same personal merit may be attended with similar increase of fortune; and for want of real anxieties, the mind becomes torpid, or bent on ambitious projects which never can be realized. We have offered these remarks, not merely as connected with morals, but as leading to practical improvement; for under all these circumstances, the remedy is to find employment for the mind in the pursuit of objects, which may lead to emulation without exciting ambition.

The last Section is on prevention. This contains every useful direction, but nothing particularly new. We much regret that the Author has given us no account of his own valuable mode of treatment, which, from its success, must have some advantages. We cannot cannot expect any thing complete on such a subject; but whatever may add to our knowledge in so melancholy a disease, ought not to be withheld from the public.

